# Classification of Atretic Small Antral Follicles in the Human Ovary

**DOI:** 10.3390/ijms242316846

**Published:** 2023-11-28

**Authors:** Fu Wei, Xueying Fan, Julieta S. del Valle, Joyce D. Asseler, Lotte E. van der Meeren, Hui Cheng, Bernard A. J. Roelen, Leoni A. Louwe, Gonneke S. K. Pilgram, Lucette A. J. van der Westerlaken, Norah M. van Mello, Susana M. Chuva de Sousa Lopes

**Affiliations:** 1 Department of Anatomy and Embryology, Leiden University Medical Center, 2333 ZC Leiden, The Netherlands; f.wei@lumc.nl (F.W.); x.fan@lumc.nl (X.F.); j.s.del_valle@lumc.nl (J.S.d.V.);; 2 Department of Obstetrics and Gynaecology, Amsterdam University Medical Center, 1105 AZ Amsterdam, The Netherlands; j.d.asseler@amsterdamumc.nl (J.D.A.); n.vanmello@amsterdamumc.nl (N.M.v.M.); 3 Amsterdam UMC, Centre of Expertise on Gender Dysphoria, 1081 HV Amsterdam, The Netherlands; 4 Amsterdam Reproduction and Development Research Institute, 1081 HV Amsterdam, The Netherlands; 5 Department of Pathology, Leiden University Medical Center, 2333 ZC Leiden, The Netherlands; l.van_der_meeren@lumc.nl; 6 Department of Pathology, Erasmus Medical Center, 3015 GD Rotterdam, The Netherlands; 7 Anatomy and Physiology, Department Clinical Sciences, Faculty of Veterinary Medicine, Utrecht University, Yalelaan 1, 3584 CL Utrecht, The Netherlands; b.a.j.roelen@uu.nl; 8 Department of Gynaecology, Leiden University Medical Center, 2333 ZA Leiden, The Netherlands; l.a.louwe@lumc.nl (L.A.L.); g.s.k.pilgram@lumc.nl (G.S.K.P.); l.a.j.van_der_westerlaken@lumc.nl (L.A.J.v.d.W.); 9 Department for Reproductive Medicine, Ghent University Hospital, 9000 Ghent, Belgium

**Keywords:** human ovary, small antral follicle, follicular atresia, classification criteria, macrophage

## Abstract

The reproductive lifespan in humans is regulated by a delicate cyclical balance between follicular recruitment and atresia in the ovary. The majority of the small antral follicles present in the ovary are progressively lost through atresia without reaching dominance, but this process remains largely underexplored. In our study, we investigated the characteristics of atretic small antral follicles and proposed a classification system based on molecular changes observed in granulosa cells, theca cells, and extracellular matrix deposition. Our findings revealed that atresia spreads in the follicle with wave-like dynamics, initiating away from the cumulus granulosa cells. We also observed an enrichment of CD68+ macrophages in the antrum during the progression of follicular atresia. This work not only provides criteria for classifying three stages of follicular atresia in small antral follicles in the human ovary but also serves as a foundation for understanding follicular degeneration and ultimately preventing or treating premature ovarian failure. Understanding follicular remodeling in the ovary could provide a means to increase the number of usable follicles and delay the depletion of the follicular reserve, increasing the reproductive lifespan.

## 1. Introduction

The human ovary is a dynamic endocrine and exocrine organ that undergoes profound changes during the menstrual cycle. In addition to the cyclic secretion of female reproductive hormones such as estrogen and progesterone, the ovary houses the entire reserve of follicles and associated somatic niche that leads to the cyclic release of mature oocytes [1].

Most follicles in the adult human ovary are dormant primordial follicles, composed of one oocyte surrounded by a single layer of flat granulosa cells and separated from the ovarian stromal compartment by a collagen-rich basement membrane. In addition, a group of small antral follicles of < 5 millimeters (mm) in diameter can be distinguished in the adult ovary at various stages of atresia (or degeneration) [1,2,3,4].

Different stages of follicular atresia have been described for several farm animals, such as ungulates, and small mammals, such as rodents [3,5,6,7,8]. However, the duration of the phases of the menstrual/oestrous cycle and associated ovarian phases (luteal and follicular) is species-specific; hence, it remains unclear how similar the process of atresia is between different mammalian species [9,10]. Most studies portray atresia of antral follicles in the human ovary as a complex process that involves the initial death of granulosa cells, characterized by the presence of pyknotic nuclei, followed by their detachment into the antrum [5,11,12]. The association between granulosa cell death and follicular atresia has prompted extensive investigations into the mechanism underlying granulosa cell death, in particular via apoptosis, which results in fragmentation of genomic DNA [13,14]. In addition to granulosa cell death, theca cell death during follicular atresia has been reported in various species, such as bovine [15], rabbit [16], and rat [17]. In contrast to the obvious apoptosis in oocytes in atretic unilaminar and preantral follicles, the oocyte within an atretic antral follicle appears to retain a normal morphology for a period of time [18].

The dynamic behavior of theca cells and granulosa cells during human follicular atresia remains poorly characterized and would benefit from further understanding and classification. In the present study, we aimed to provide a comprehensive examination of the morphological characteristics of different atretic follicles in the human ovary, along with an analysis of molecular changes occurring in the different types of follicular cells, including granulosa and theca cells, as well as extracellular matrix proteins and CD68+ macrophages during atresia. Based on our findings, we established robust classification criteria to distinguish three levels of atresia in human small antral follicles.

## 2. Results

### 2.1. Characterization of Granulosa Cells in Type 1 Atretic Follicles in Adult Ovaries

**Figure 1:**
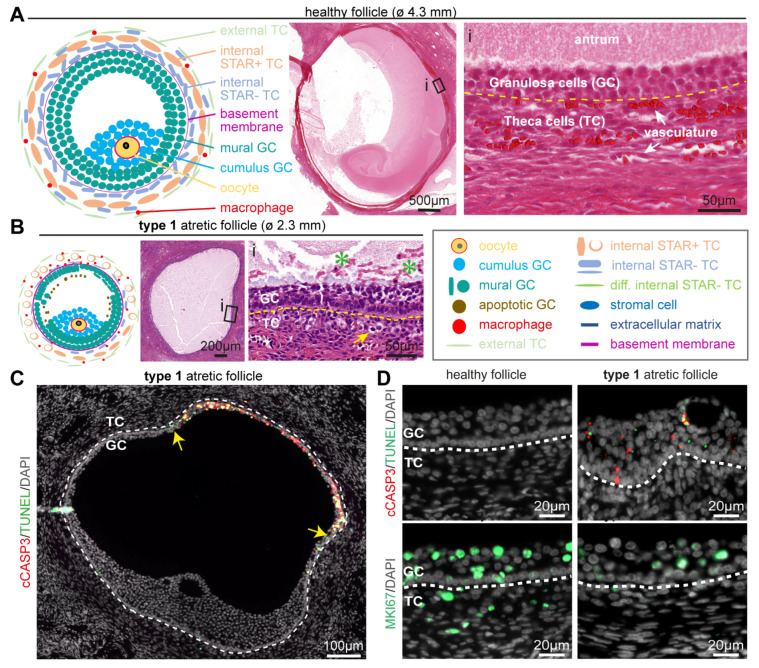
Follicular cell types in healthy small antral follicles and type 1 atretic follicles. (**A**) Overview of the main follicular cell types, such as granulosa cells (GC) and theca cells (TC), and the follicular structure of healthy small antral follicles. Yellow dashed line in the magnified hematoxylin-eosin-stained inset (i) depicts the basement membrane. (**B**) Overview of the main follicular cell types and structures in type 1 atretic follicles. In the magnified hematoxylin-eosin-stained inset: yellow arrow points to a TC with hyaline area; green asterisks in the antral cavity mark GC showing pyknotic nuclei; and yellow dashed line marks the basement membrane. (**C**) Immunofluorescence for cCASP3 and TUNEL in type 1 atretic follicle from cis female donors. Yellow arrows indicate the boundary of apoptotic mural GC. White dashed line depicts the basement membrane. (**D**) Immunofluorescence for cCASP3, TUNEL and MKI67 in healthy small antral follicles and type 1 atretic follicles from cis female donors. White dashed line depicts the basement membrane.

Healthy small antral follicles presented different cell types, including the oocyte, two types of granulosa cells (mural and cumulus), and a collagen-rich basement separating the follicle from the stromal compartment [19] (Figure 1A). The stromal compartment, in particular in the vicinity of the follicular basement membrane, is a complex and dynamic mixture of cells, collectively known as theca cells, that change identity as the follicle grows, together with macrophages and vasculature [20]. The theca cells develop to form a layer of fibroblast-like external theca cells and a layer of internal theca cells that can be divided into steroidogenic (STAR+) and non-steroidogenic (STAR−) theca cells [21,22] (Figure 1A).

Representing the earliest stage of atresia, in type 1 atretic small antral follicles mural granulosa cells were still observed covering the entire inner perimeter of the basement membrane, but the granulosa cells in contact with the basement membrane became more columnar. In addition, the inner mural granulosa cells started to detach to the follicular fluid and showed pyknotic nuclei. The basement membrane thickened and the internal theca cells started to round up and showed an increased (hyaline) cytoplasmic area (Figure 1B).

**Figure 2:**
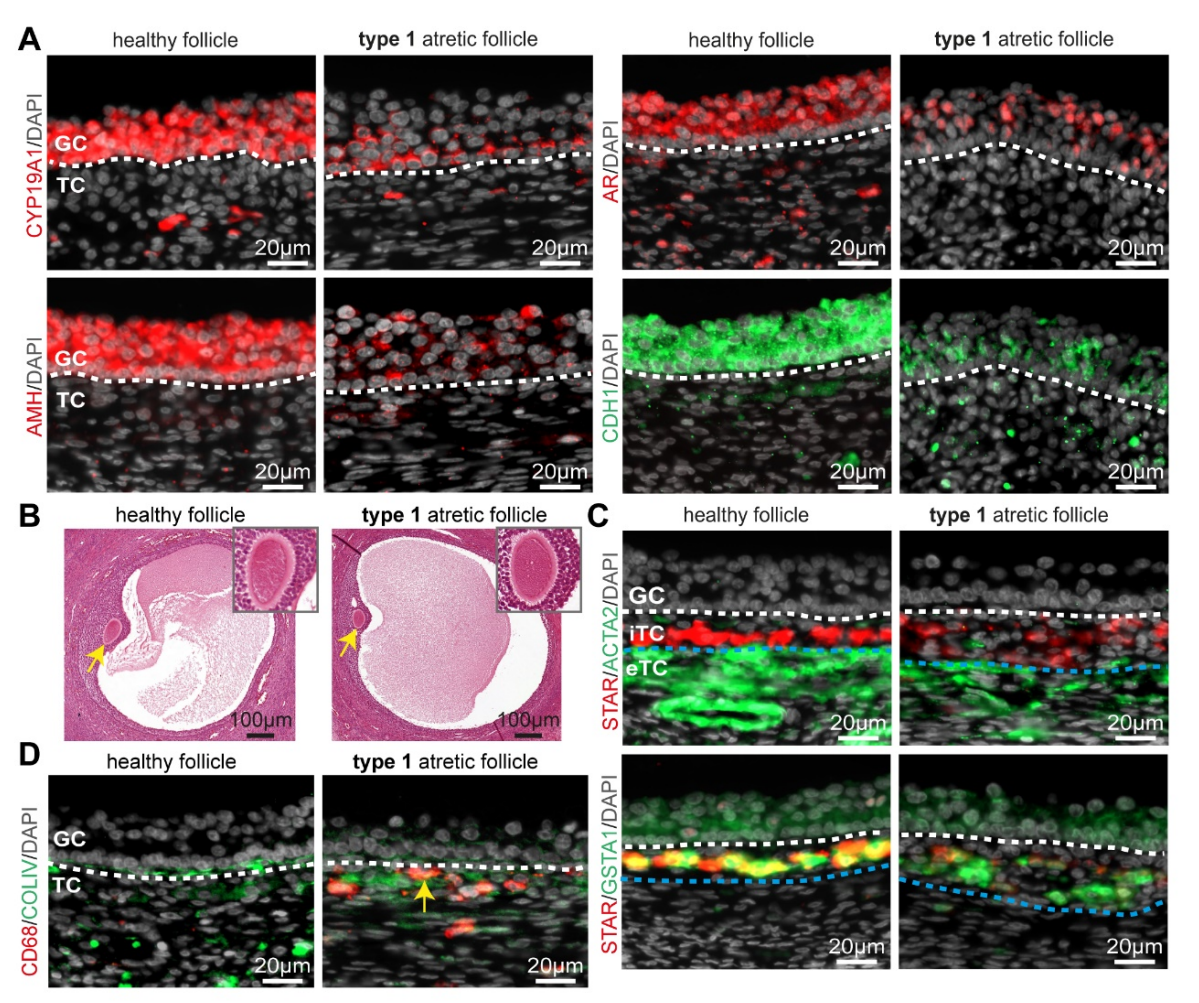
Granulosa and theca cells in healthy small antral follicles and type 1 atretic follicles from cis female donors. (**A**) Immunofluorescence for CYP19A1, AMH, AR, and CDH1 in healthy small antral follicles and type 1 atretic follicles from cis female donors. White dashed line depicts the basement membrane. (**B**) Histological sections (hematoxylin-eosin-stained) of adult ovaries from trans masculine donors depicting a healthy small antral follicle and type 1 atretic follicle containing the oocyte (yellow arrow), magnified in the top-right part. (**C**) Immunofluorescence for STAR and ACTA2 (top panels) and STAR and GSTA1 (bottom panels) in healthy small antral follicles and type 1 atretic follicles from cis female donors. White dashed line depicts the basement membrane between granulosa cells (GC) and internal theca cells (iTC); blue dashed line depicts the border between iTC and external theca cells (eTC). (**D**) Immunofluorescence for CD68 and COLIV in healthy small antral follicles and type 1 atretic follicles from cis female donors. Yellow arrow points to CD68+ macrophages. White dashed line depicts the basement membrane.

The most prominent difference between type 1 atretic follicles and healthy follicles was the degeneration of the mural granulosa cells (Figure 1C). The mural granulosa cells staring from the opposite side to the cumulus region showed increased apoptosis, as evidenced by a strong TUNEL signal in clusters of cells in the antrum and granulosa cells expressing cleaved Caspase3 (cCASP3) in adult cis females (Figure 1C,D) and trans masculine small antral follicles (Figure S1A). Moreover, decreased proliferation was evidenced by lower numbers of mural granulosa cells and theca cells that were positive for MKI67 (or mKi67) in adult cis females (Figure 1D) and trans masculine small antral follicles (Figure S1A). In type 1 atretic follicles, the mural granulosa cells also showed a pronounced downregulation of cell-cell adhesion molecule Cadherin 1 (CDH1) and lower expression of several markers of granulosa cells, such as aromatase CYP19A1 responsible for the conversion of androgens to estrogens [23], anti-Müllerian hormone (AMH) [24], and androgen receptor (AR) [25] in adult cis female (Figure 2A) and trans masculine small antral follicles (Figure S1A). The pronounced downregulation of CYP19A1 suggested compromised estrogen synthesis.

### 2.2. Dynamics in the Theca Cell Layer and Oocyte in Type 1 Atretic Follicles in Adult Ovaries

We observed morphologically comparable oocytes in type 1 and healthy follicles (Figure 2B). However, both theca cell layers (interna and externa) showed considerable differences between healthy and type 1 atretic follicles. We observed a decreased expression of STAR in the steroidogenic internal theca cells (iTC) as well as a decrease in ACTA2 (or smooth muscle actin alpha 2) in the external theca cells (eTC) in type 1 atretic follicles in adult cis females (Figure 2C) and trans masculine small antral follicles (Figure S1B). A decrease in STAR expression suggested a decreased production of androgens [26]. Interestingly, the levels of glutathione S-transferase alpha 1 (GSTA1), another enzyme involved in steroidogenesis [27], expressed in mural granulosa cells, and steroidogenic iTC [21] remained similar between healthy and type 1 atretic follicles in adult cis female (Figure 2C) and trans masculine small antral follicles (Figure S1B).

Macrophages are known to play a phagocytotic role by removing cell debris after apoptosis [28], hence we also investigated the presence of CD68+ macrophages in type 1 atretic follicles. In contrast to healthy follicles that did not contain CD68+ macrophages within the theca cell layers, we observed that CD68+ macrophages were recruited to the iTC of type 1 atretic follicles in adult cis female (Figure 2D) and trans masculine small antral follicles (Figure S1C). In areas with a non-continuous COLIV+ basement membrane containing a reduced number of attached mural granulosa cells in type 1 atretic follicles, CD68+ macrophages were observed in the antral cavity (Figure S1C).

### 2.3. ACTA2+ Fibroblast-like Cells Coat the Antrum in Type 2 Atretic Follicles in Adult Ovaries

**Figure 3:**
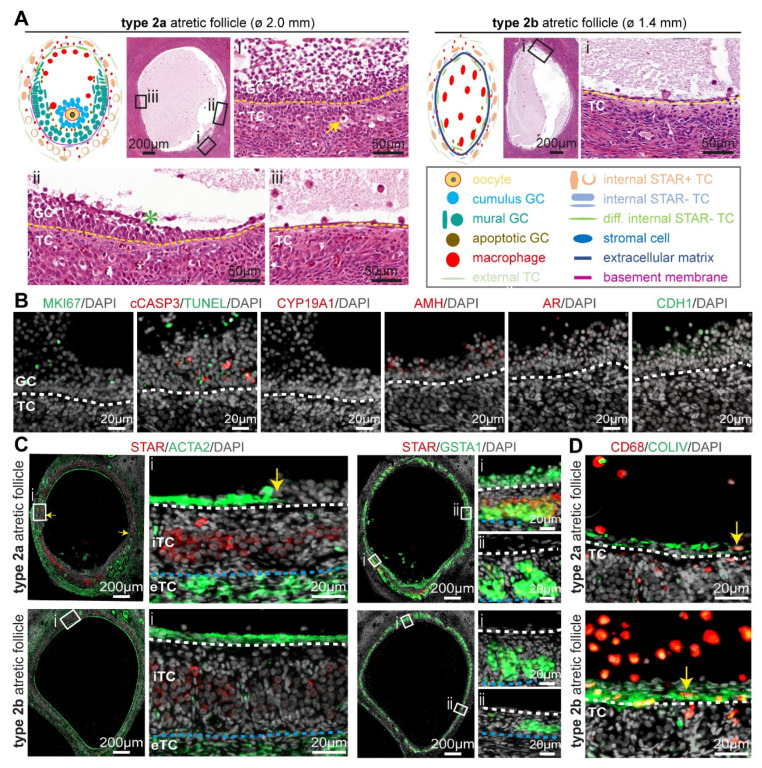
Type 2 atretic follicles in human ovaries. (**A**) Overview of the main follicle-associated cell types, including granulosa cells (GC) and theca cells (TC), and the structure of type 2 (2a and 2b) atretic follicles. In the magnified hematoxylin-eosin-stained areas: yellow arrow points to a TC with hyaline area; green asterisk in antral cavity marks GC showing pyknotic nuclei; and yellow dashed line marks the basement membrane. (**B**) Immunofluorescence for MKI67, CYP19A1, AR, AMH, CDH1, and type 2a atretic follicles from cis female donors. White dashed line depicts the basement membrane. (**C**) Immunofluorescence staining for STAR and ACTA2 (left panels) and STAR and GSTA1 (right panels) in type 2 atretic follicles from cis female donors. In the magnified areas: yellow arrow points to the transition between ACTA2+ and ACTA2-areas bordering the antral cavity; white dashed line depicts the basement membrane; blue dashed line depicts the border between internal theca cells (iTCs) and external theca cells (eTC). (**D**) Immunofluorescence for CD68 and COLIV in healthy small antral follicles and type 2 atretic follicles from cis female donors. Yellow arrows point to CD68+ macrophages. White dashed line depicts the basement membrane.

In type 2 atretic follicles, the basement membrane either included the area of cumulus cells still loosely attached (type 2a) or contained no granulosa cells attached (type 2b) (Figure 3A), depending on how the tissue is sectioned. We classified these two subtypes of type 2 atretic follicles based on the presence of the cumulus area, while the mural granulosa cells were present in both subtypes. Due to this morphological feature, type 2a atretic follicles appear strongly asymmetrical, as the granulosa cells in the inner perimeter of the basement membrane are progressively replaced by a few layers of flat fibroblast-like cells in the region away from the cumulus area (Figure 3A).

The granulosa cells in type 2 atretic follicles continued to degenerate, as indicated by TUNEL and cCASP3 in adult cis female (Figure 3B) and trans masculine small antral follicles (Figure S2A). The granulosa cells also showed loss of CYP19A1 and CDH1, while retaining low levels of AR, AMH, and MKI67 in adult cis females (Figure 3B) and trans masculine small antral follicles (Figure S2B), comparable to those observed in type 1 atretic follicles.

Notably, in the part of the follicle without cumulus granulosa cells, the flat fibroblast-like cells in the inner perimeter of the basement membrane were strongly positive for ACTA2 in adult cis female (Figure 3C) and trans masculine small antral follicles (Figure S2C). In addition, a sharp border was present between flat ACTA2+-positive cells and the granulosa cells at the basement membrane bordering the antral cavity (yellow arrows in Figure 3C and Figure S2C). The steroidogenic iTC expressed GSTA1 throughout the entire follicular perimeter, but STAR expression was significantly decreased in the iTC in the part of the follicle where the granulosa cells were replaced by the flat ACTA2+ cells (Figure 3C and Figure S2C). In type 2 atretic follicles, we detected a higher abundance of CD68+ macrophages in the antral cavity in the proximity of the flat (COLIV-rich) fibroblast-like cells (Figure 3B and Figure S2C).

### 2.4. In Type 3 Atretic Follicles the Antral Cavity Filled and the Oocyte Degenerated

**Figure 4:**
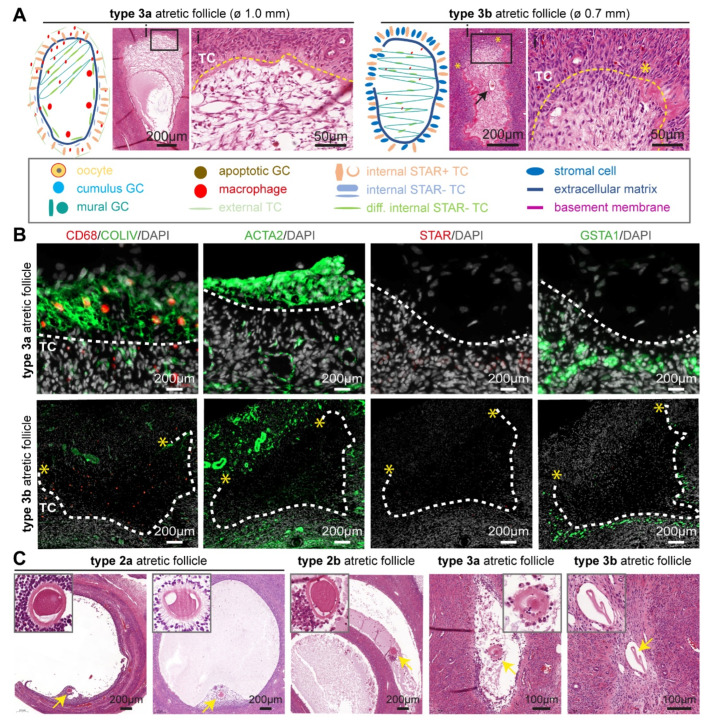
Type 3 atretic follicles in human ovaries. (**A**) Overview of the main follicle-associated cell types, such as granulosa cells (GC) and theca cells (TC), and follicular structure in type 3 (3a and 3b) atretic follicles from adult ovaries. In the magnified hematoxylin-eosin-stained areas: yellow dashed line marks the place of the basement membrane; yellow asterisks mark the place where the basement membrane was ruptured; and black arrow points to the remnant of the antral cavity. (**B**) Immunofluorescence for CD68, COLIV, ACTA2, STAR, and GSTA1 in type 3 atretic follicles from cis female donors. White dashed line marks the basement membrane. Yellow asterisks mark the place where the basement membrane was ruptured. (**C**) Histological sections (haematoxylin-eosin-stained) of adult ovaries depicting type 2 and type 3 atretic follicles containing the oocyte or oocyte remnants (yellow arrow), magnified in the top part.

In type 3 atretic follicles, the antral cavity of the degenerating small antral follicle was progressively filled with a mesh of flat fibroblast-like cells. From single paraffin sections, it was difficult to determine whether the antral cavity was completely filled; therefore, we differentiated between two subtypes of type 3 atretic follicles: type 3a showed strong asymmetry and a visible antral cavity partially filled by multiple layers of a loose mesh of connective tissue, whereas in type 3b atretic follicles, the connective tissue filled the entire antral cavity (Figure 4A). In type 3 follicles, the basement membrane continued to thicken but was often interrupted in one large area (Figure 4A).

The connective tissue filling the antral cavity showed strong expression of ACTA2 and COLIV, while their expression is downregulated upon closure in adult cis female (Figure 4B) and trans masculine small antral follicles (Figure S3A). Moreover, the surrounding steroidogenic iTC continued to express GSTA1 in adult cis females (Figure 4B) and trans masculine small antral follicles (Figure S3A). The CD68+ macrophages appeared to be retained within the connective tissue in type 3 atretic follicles in adult cis females (Figure 4B) and trans masculine small antral follicles (Figure S3A).

In contrast to type 2 atretic follicles that seemed to retain the oocyte, the oocyte in type 3 atretic follicles appeared degenerated, and in type 3b atretic follicles, an empty acellular structure, possibly the remaining of the zona pellucida, was visible (Figure 4C).

### 2.5. Quantification of Atretic Human Small Antral Follicles

Similar to the atretic small antral follicles observed in the ovaries of adult cis females (n = 6 donors) and trans masculine donors (n = 27) (Table S1), we also confirmed the presence of three morphologically distinguishable types of atretic small antral follicles in the ovary of a cis female carrying a *BRCA1* mutation (Figure S3B). In that ovary, follicular degeneration was also more pronounced in the region distal to the cumulus granulosa in type 2a atretic follicles (Figure S3B).

**Figure 5:**
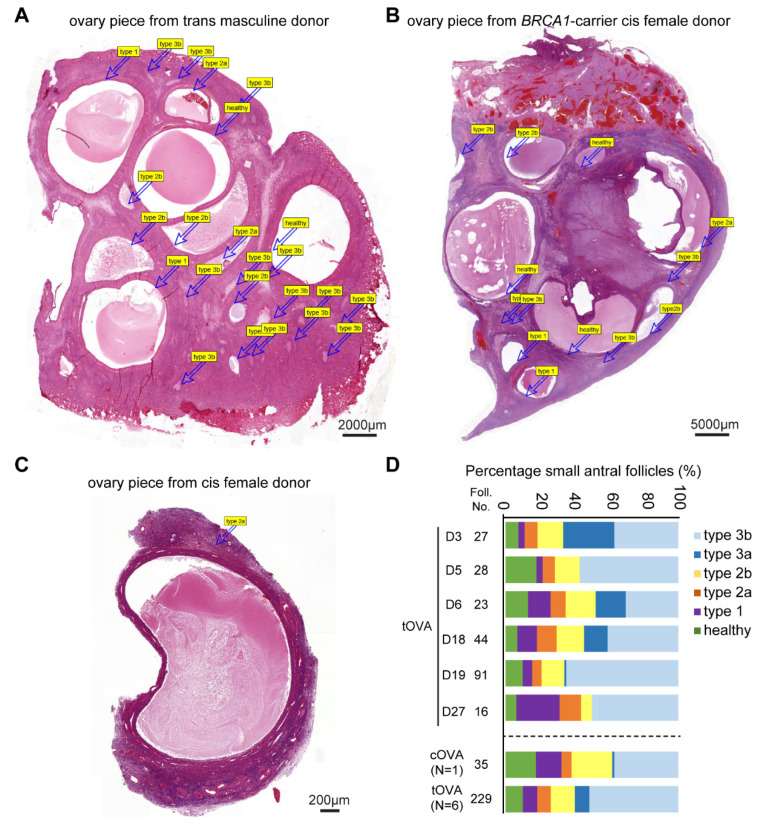
Quantification of healthy and atretic small antral follicles. (**A**) Overview of haematoxylin-eosin-stained ovarian piece from a trans masculine donor. The labels correspond to distinct follicles. (**B**) Overview of haematoxylin-eosin-stained ovarian piece from a *BRCA1*-carrier cis female donor. The labels correspond to distinct follicles. (**C**) Overview of haematoxylin-eosin-stained ovarian fragment from a cis female donor. The labels correspond to distinct follicles. (**D**) Quantification of atresia in small antral follicles in ovaries of several trans masculine donors (**D**) separately and pooled (tOVA) as well as in ovaries of one cis female *BRCA1*-carrier donor (cOVA).

Access to relatively large histological sections of ovaries from trans masculine donors (n = 6) and the cis female carrying a *BRCA1* mutation (n = 1) (Figure 5A,B), compared with the isolated follicles of cis female donors undergoing fertility preservation (Figure 5C), allowed us to perform quantification of small antral follicles undergoing atresia (Figure 5D). The quantification revealed that the majority (about 80%) of the small antral follicles present in the ovaries analyzed (n = 7) were undergoing atresia, and from those, most were type 3b atretic follicles (Figure 5D).

### 2.6. Morphological and Molecular Characteristics of Atretic Small Antral Follicles

Overall, the morphological changes observed during atresia of human small antral follicles showed similarities with those reported in mammals, such as bovine [5,29], caprine [30], rat [6], mouse [31], and guinea pig [8] (Table S2).

Based on our analysis, we summarized characteristics to differentiate small antral follicles in different stages of atresia (Table 1). Healthy follicles had a well-defined layer of mural granulosa cells (average 4.2 cell layer thick) with a low apoptotic index (1.1%) and a high mitotic index (31.5%), surrounded by an intact basement membrane and an iTC layer of ellipsoidal-shaped cells with an average of 1.9 cell layer thick. Type 1 atretic follicles contained a thinner mural granulosa cell layer (average 3.4 cell layer thick), with a higher apoptotic index (21.9%) and a lower mitotic index (14.4%), but a thicker iTC layer with round hyaline cells (average 3.7 cell layer thick). Type 2 atretic follicles were characterized by an irregular and thinner mural granulosa cell layer with a high apoptotic index ranging from 17.3–78.6% and a low mitotic index ranging between 4.6% and 0%, and the basement membrane was not intact. Type 3 atretic follicles no longer contain an oocyte, and the follicular cavity is filled with connective tissue. In addition to morphological parameters, the expression level of specific markers used to visualize specific cell types in the follicle can also be used to differentiate small antral follicles in different stages of atresia (Table 2).

## 3. Discussion

The vast majority of follicles in the human ovary undergo atresia, but our understanding of this process remains limited. Increasing our knowledge on the regulation of this process could aid in the preservation of the follicular reserve and postpone exhaustion of the ovary. In the present study, we established classification criteria for human small antral follicle atresia based on their morphological and molecular characteristics. During the early stages of atresia (type 1), the mural granulosa cells bordering the antral cavity become loose and pyknotic (antral atresia). In bovine ovaries, a different type of early follicular atresia has also been described, where the most basal granulosa cells degenerate while granulosa cells in contact with the antral cavity remain healthy (basal atresia) [11,12], a phenomenon not observed in our current study in humans. During the intermediate stage (type 2), the granulosa cells detached from the basement membrane and were removed from the follicle. In the final stage (type 3), the atretic follicles reduced in size, and fibroblast-like cells gradually filled the narrowing antral cavities.

Granulosa cell death is a well-known process during follicular atresia of antral follicles that occurs primarily via apoptosis [4,11,14]. Our study confirmed apoptosis (cCASP3 and TUNEL) in mural granulosa cells in type 1 atretic follicles. In addition, type 1 atretic follicles showed a decrease in CYP19A1, AMH, AR, CDH1, and MKI67 expression in granulosa cells with normal morphology. This suggested that using a combination of these markers could be used to distinguish early (type 1) atretic follicles from healthy follicles. Several studies on animals have also reported a reduced expression of the aromatase CYP19A1 in atretic follicles [32,33]. Our findings suggested that this reduction occurred rapidly at an early stage (type 1), indicating an early loss of estrogen synthesis in granulosa cells. This loss hinders granulosa cell proliferation and may impair the activity of AR and AMH in promoting antral follicular growth [24,25], ultimately contributing to its degeneration. Additionally, reduced CDH1-mediated cell-cell adhesion and disrupted gap junctions, observed using electron microscopy in mice [34], accounted for the detachment of granulosa cells, causing further degeneration.

While granulosa cells have been extensively studied during ovarian follicular atresia, the role of theca cells has received less attention. In type 1 atretic follicles, the iTC displayed a rounder shape with large hyaline areas, which can serve as a reference feature for identifying early follicular atresia. Furthermore, the expression of STAR, a marker of steroidogenesis, was strongly downregulated in iTC already in type 1 atretic follicles, in contrast with the stable expression of GSTA1 in steroidogenic iTC during atresia. Reduced expression of STAR in steroidogenic iTC may impair the transport of cholesterol and the downstream synthesis of testosterone [26]. Impaired cholesterol transport may lead to its accumulation, possibly giving rise to the hyaline areas in the cell. Testosterone produced by steroidogenic iTC can be delivered to granulosa cells to synthesize estrogen and maintain their growth [35]. Thus, it is possible that reduced testosterone synthesis in steroidogenic iTC may trigger or accelerate granulosa cell degeneration.

Unlike steroidogenic iTC, eTC are smooth-muscle-like cells that play an important role in supporting the spherical shape of the growing follicle by providing extracellular matrix [26]. We noticed that atretic follicles tend to lose their spherical shape and collapse during the type 2 stage, when a pronounced decrease in ACTA2 is observed in the eTC. Indeed, since ACTA2 is positively associated with extracellular matrix production and stiffness in several tissues [36,37], the decrease of ACTA2 in the eTC may be responsible for the observed follicular collapse. Interestingly, a population of fibroblast-like cells expressing relatively high levels of ACTA2 populated the inner perimeter of the basement membrane in type 2 atretic follicles and gradually filled the antral cavity. We speculate that these cells originate from non-steroidogenic iTC bordering the basement membrane. Our hypothesis is supported by two observations: first, there was no evidence of iTC cell death; and second, the ACTA2+ cells were always surrounded by a layer of GSTA1+ steroidogenic iTC. Although the involvement of non-steroidogenic iTC in the process of atresia remains to be determined, we demonstrate that the dynamics of ACTA2 expression are helpful biomarkers in determining the different stages of follicular atresia.

Immune cells are known to be involved in follicle atresia [38]. In agreement, we observed the recruitment of CD68+ macrophages at the onset of follicular atresia (type 1) and showed that they remained present until late atresia (type 3). In type 1 atretic follicles, CD68+ macrophages were present in the iTC layer and penetrated into the antral cavity as the COLIV+ basement membrane became discontinuous. Prior to the presence of macrophages in the antral cavity, neighboring granulosa cells may help clear the degenerating granulosa cells, as described in rodents [34,39]. We speculate that CD68+ macrophages entering the antral cavity exhibit phagocytic clearance functions, whereas during late atresia they may acquire restorative functions, in line with observations of macrophages exhibiting different phenotypes in different environments [40,41]. Further investigation is necessary to explore the presence of additional immune cells during the process of follicular atresia in humans.

Our study has several limitations. Our classification criteria were developed using ovaries from both cis females (n = 7) and trans masculine donors (n = 27). The donor population was heterogeneous regarding age, duration of hormonal treatment, and type of hormonal treatment, but the large number of donors (n = 34) allowed us to determine and characterize the main cell types associated with the different stages of atresia in small antral follicles. Moreover, the ovaries analyzed from trans masculine donors and *BRCA1*-carrier were intact, enabling us to observe the complete spectrum of follicles in a single histological section and allowing robust quantification. By contrast, ovarian tissue available from cis female donors undergoing fertility preservation was fragmented, and each fragment typically contained just 1–2 small antral follicles (Figure 5A–C), allowing measurements but not adequate for quantification of follicles. Our classification criteria did not take the molecular characterization of the degenerating oocyte into account, as due to the size difference between the oocyte and the small antral follicle, oocytes were mostly absent from the paraffin sections analyzed. Finally, atresia also occurs in pre-antral follicles and large antral follicles, but those were not taken into account in the present study.

In conclusion, our study proposes classification criteria for human follicular atresia based on the morphological and molecular characteristics of the different somatic cell types associated with the follicle (granulosa cells, theca cells, and immune cells). Our findings not only enhance our understanding of human follicular degeneration, providing a broader insight into the complex process of follicular atresia, but can also prove beneficial to improve research on infertility and ovarian disorders, such as premature ovarian failure.

## 4. Materials and Methods

### 4.1. Study Population and Tissue Collection

This study included ovarian tissue from: 27 trans masculine donors who underwent gender affirming oophorectomy at the Amsterdam University Medical Center and were on gender-affirming hormone treatment with testosterone for at least 1 year; 1 *BRCA1*-carrier cis female who underwent prophylactic oophorectomy at the Leiden University Medical Center; and ovarian tissue from 6 cis female donors collected from rest material from ovarian medulla/inner cortex tissue after the ovaries, collected by elective ovariectomy, was used for fertility preservation purposes at the Leiden University Medical Center (Table S1). After surgery, the ovaries of trans masculine donors and the ovarian medulla fragments of cis female donors that remained after fertility preservation procedures were kept in ice-cold saline solution and transferred to the laboratory at Leiden University Medical Center within 1–2 h.

### 4.2. Histology

Freshly collected ovaries were cut into several pieces and fixed in 4% paraformaldehyde (Merck, Darmstadt, Germany) in PBS overnight at 4 °C. The tissue pieces were washed 3 × 30 min with PBS, transferred to 70% ethanol, and embedded in paraffin using a Shandon Excelsior tissue processor (Thermo Fisher Scientific, Waltham, MA, USA). Following embedding, the tissue was sectioned (5 μm) with an RM2065 microtome (Leica Instruments GmbH, Wetzlar, Germany) and pasted onto StarFrost microscope slides (3057-1, Waldemar Knittel, Braunschweig, Germany). Staining with hematoxylin and eosin (H&E) was performed on the sections using a standard protocol. In the present study, H&E staining was performed on 69 sections from 34 donors (39 sections from 27 trans masculine donors, 3 sections from 1 *BRCA1*-carrier cis female donor, and 27 sections from 6 cis female donors undergoing fertility preservation).

### 4.3. Immunofluorescence

Tissue sections were deparaffinized in xylene (Merck), rehydrated in ethanol using a sequential dilution, and finally rinsed in distilled water. The sections were then treated with Tris-EDTA buffer (10 mM Tris, 1mM EDTA solution, pH 9.0) and heated for 12 min at 98 °C in a microwave (TissueWave 2 Microwave, Thermo Fisher Scientific). After cooling down, the sections were washed with PBS (2 × 5 min) and with 0.05% Tween-20 (822184, Merck) in PBS (PBST) (5 min). To prevent non-specific binding of antibodies, tissue sections were incubated with 1% BSA (A8022-100G, Life Technologies, Eugene, OR, USA) in PBST (blocking solution) for 1 h at room temperature (RT), followed by overnight incubation at 4 °C with primary antibodies diluted in blocking solution. The primary antibodies used were mouse anti-CYP19A1 (1:100, sc-374176, Santa Cruz, Dallas, TX, USA), mouse anti-AR (1:100, sc-7305, Santa Cruz), mouse anti-AMH (1:100, MCA2246T, BioRad, Hercules, CA, USA), rabbit anti-CDH1 (1:100, CST3195S, Cell Signaling, Danvers, MA, USA), rabbit anti-MKI67 (1:100, ab15580, Abcam, Cambridge, UK), rabbit anti-cCASP3 (Asp175) (1:100, CST9661S, Cell Signaling), rabbit anti-COLLIV (1:100, AB748, Merck), mouse anti-CD68 (1:100, M087629-2, DAKO, Glostrup, Denmark), rabbit anti-ACTA2 (1:100, ab5694, Abcam), mouse anti-STAR (D-2) (1:100, sc-166821, Santa Cruz), rabbit anti-GSTA1 (1:100, HPA053817, Atlas Antibodies, Bromma, Sweden). Thereafter, tissue sections were rinsed with PBS (2 × 5 min), and PBST (5 min) and incubated with secondary antibodies and 4′,6-diamidino-2-phenylindole (DAPI) (1:500, Life Technologies) in blocking solution for 1 h at RT. The secondary antibodies used were Alexa Fluor 488 donkey anti-rabbit IgG (1:500, A-21206, Life Technologies), Alexa Fluor 594 donkey anti-mouse IgG (1:500, A-21203, Life Technologies), and Alexa Fluor 647 donkey anti-goat IgG (1:500, A-21447, Life Technologies). For TUNEL staining, we used the In Situ Cell Death Detection Kit (FITC) (11684817910, Roche, Mannheim, Germany) following the manufacturer’s protocol. ProLong Gold (P36930, Life Technologies) was used for section mounting.

### 4.4. Imaging

The H&E-stained slides were scanned using a Panoramic 250 digital scanner (3DHISTECH, Budapest, Hungary) and viewed with CaseViewer v2.4.0 (3DHISTECH, Budapest, Hungary). The immunofluorescence-stained slides were scanned using a ZEISS Axioscan 7 digital scanner (ZEISS, Jena, Germany) and viewed with ZEN v3.3.0 (ZEISS). Confocal fluorescence images were acquired with a TC SP8 inverted confocal microscope (Leica), using an ×40 oil immersion objective and LAS X v3.7.4 (Leica). Figures were assembled using Adobe Illustrator v25.2.3 (Adobe, San Jose, CA, USA).

### 4.5. Follicular Measurements and Quantification

We identified 503 small antral follicles (<5 mm) in ovarian sections of 34 donors and investigated morphological features such as the shape of the follicles, the morphology of granulosa cells, internal theca cells and oocytes, and the integrity of the basal membrane (Table 1). Using the built-in ruler tool in CaseViewer v2.4.0 software (3DHISTECH, Budapest, Hungary), we measured the follicle diameter, thickness, and number of cell layers in the mural granulosa cells (averaged from the thinnest and thickest regions). We also calculated the mitotic index (MKI67-positive cells) and apoptotic index (TUNEL-positive cells) in three follicles (n = 3) per follicular type. We used the line tool in ZEN v3.3.0 software (Zeiss, Köln, Germany) to measure the thickness and number of cell layers in the GSTA1+ theca cells (averaged from the thinnest and thickest regions). To quantify the different types of atretic small antral follicles, we used six transmasculine and one *BRCA1*-carrier donor, each with three randomly selected H&E-stained slides from different parts of the ovary. Based on the morphological criteria described in this study, we counted and classified all small antral follicles (<5 mm) in those slides into: healthy follicles, type 1 atretic follicles, type 2 (2a of 2b) atretic follicles, and type 3 (3a or 3b) atretic follicles.

## Figures and Tables

**Table 1:** Morphological characteristics of atretic small antral follicles.

Tissue Features of Small Antral Follicles		Healthy	Type 1	Type 2		Type 3	
				Type 2a	Type 2b	Type 3a	Type 3b
Follicle	total number of follicles analysed	47	59	49	72	51	225
	shape of follicle	round/ellipsoid	round/ellipsoid	ellipsoid/irregular	ellipsoid/irregular	irregular	irregular
	size of follicle ± SD (cm) (size range)	2.2 ± 1.3 (0.8–5.0)	2.0 ± 0.8 (0.9–4.3)	1.6 ± 0.7 (0.5–3.6)	1.2 ± 0.7 (0.15–2.7)	0.5 ± 0.4 (0.08–2.1)	0.2 ± 0.1 (0.02–0.25)
Mural Granulosa Cells (mGC)	number of mGC layers ± SD (number range)	4.2 ± 1.1 (2–10)	3.4 ± 1.1 (1–9)	2.2 ± 0.9 (0–8)	0	0	0
	thickness of mGC ± SD (μm) (size range)	32.5 ± 12.0 (10.0–91.1)	25.5 ± 10.4 (4.0–89.0)	16.5 ± 7.2 (0.0–75.0)	0	0	0
	shape of mGC in contact with BM	round	round/columnar	columnar/flat	n/a	n/a	n/a
	apoptotic index (% TUNEL+ mGCs) ± SD (index range)	1.1 ± 1.5 (0.0–3.9)	21.9 ± 10.1 (6.0–32.7)	17.3 ± 10.6 (6.0–38.0)	78.6 ± 5.9 (in cavity) (75.0–81.8)	0	0
	mitotic index (% MIK67+ mGCs) ± SD (index range)	31.5 ± 3.9 (26.6–40)	14.4 ± 6.8 (7.3–25)	4.6 ± 2.0 (2.3–6.7)	0	0	0
Basement Membrane (BM)	BM integrity	intact	intact	not intact	not intact	ECM deposition	ECM deposition
	thickness of BM	+	+	++	++	+++	+++
Internal Theca Cells (iTC)	number of (GSTA1+) iTC layers ± SD (number range)	1.9 ± 0.6 (1–4)	3.7 ± 1.5 (1–7)	4.8 ± 1.4 (2–10)	3.3 ± 1.1 (2–8)	2.5 ± 0.6 (2–5)	1.4 ± 1.3 (0–4)
	thickness of (GSTA1+) iTC ± SD (μm) (size range)	16.1 ± 6.0 (8.0–32.9)	34.2 ± 13.6 (8.0–75.1)	39.3 ± 16.8 (15.0–91.9)	27.9 ± 8.7 (15.0–62.3)	24.9 ± 5.0 (18.5–40.8)	12.8 ± 11.6 (0–5)
	shape of iTC	ellipsoid	round (hyaline)	round (hyaline)	round (hyaline)	elongated	elongated
	orientation of (GSTA1+) iTC relative to BM	parallel	parallel	parallel	parallel	perpendicular	perpendicular
Macrophages	accumulation	ovarian stroma	iTC layer	follicular cavity	follicular cavity	connective tissue in follicular cavity	connective tissue in follicular cavity
Oocyte	shape of zona pellucida	round	round	round/irregular	round/irregular	irregular	irregular
	presence of oocyte	yes	yes	yes	yes	no	no

Abbreviations: ECM, extra cellular matrix; SD, standard deviation; mm, micrometer; +++, high expression; ++, moderate expression; +, low expression.

**Table 2:** Expression levels of markers of interest in specific cell types in atretic small antral follicles.

Cell Type Markers Used		Healthy	Type 1	Type 2		Type 3a	
				Type 2a	Type 2b	Type 3a	Type 3b
Mural Granulosa Cells (mGC)	MKI67	+++	+	+/−	−	−	−
	cCASP3	−	++	++	++ (in cavity)	−	−
	TUNEL	−	++	++	++ (in cavity)	−	−
	CYP19A1	+++	+	−	−	−	−
	AMH	+++	+	+/−	−	−	−
	AR	++	++	+/−	−	−	−
	CDH1	+++	+	−	−	−	−
	GSTA1	++	++	+/−	−	−	−
Internal Theca Cells (iTC)	COLIV	+	++	++	++	++/−	−
	ACTA2	−	−	++/−	++	+++/+	+
	STAR	+++	++	+	+/−	+/−	+/−
	GSTA1	+++	+++	+++	+++	++	++
External Theca Cells (eTC)	ACTA2	+++	++	++/+	+	+	+
Macrophages	CD68	+/−	++	+++	+++	++	++

Abbreviations: +++, high expression; ++, moderate expression; +, low expression; −, no expression.

## Data Availability

Data is contained within the article and supplementary material.

## Supplementary Materials

The following supporting information can be downloaded at: https://www.mdpi.com/article/10.3390/ijms242316846/s1.

## References

[1] Gougeon A. (2010). Human ovarian follicular development: From activation of resting follicles to preovulatory maturation. Ann. Endocrinol..

[2] McNatty K.P., Hillier S.G., van den Boogaard A.M., Trimbos-Kemper T.C., Reichert L.E., van Hall E.V. (1983). Follicular development during the luteal phase of the human menstrual cycle. J. Clin. Endocrinol. Metab..

[3] Tilly J.L., Kowalski K.I., Johnson A.L., Hsueh A.J. (1991). Involvement of apoptosis in ovarian follicular atresia and postovulatory regression. Endocrinology.

[4] Vaskivuo T.E., Tapanainen J.S. (2003). Apoptosis in the human ovary. Reprod. Biomed. Online.

[5] Irving-Rodgers H.F., van Wezel I.L., Mussard M.L., Kinder J.E., Rodgers R.J. (2001). Atresia revisited: Two basic patterns of atresia of bovine antral follicles. Reproduction.

[6] Osman P. (1985). Rate and course of atresia during follicular development in the adult cyclic rat. J. Reprod. Fertil..

[7] Sugimoto M., Manabe N., Kimura Y., Myoumoto A., Imai Y., Ohno H., Miyamoto H. (1998). Ultrastructural Changes in Granulosa Cells in Porcine Antral Follicles Undergoing Atresia Indicate Apoptotic Cell Death. J. Reprod. Dev..

[8] Wang W., Liu H.L., Tian W., Zhang F.F., Gong Y., Chen J.W., Mao D.G., Shi F.X. (2010). Morphologic observation and classification criteria of atretic follicles in guinea pigs. J. Zhejiang Univ. Sci. B..

[9] Baerwald A.R. (2009). Human antral folliculogenesis: What we have learned from the bovine and equine models. Anim. Reprod..

[10] Baerwald A.R., Adams G.P., Pierson R.A. (2012). Ovarian antral folliculogenesis during the human menstrual cycle: A review. Hum. Reprod. Update.

[11] Glamoclija V., Vilovic K., Saraga-Babic M., Baranovic A., Sapunar D. (2005). Apoptosis and active caspase-3 expression in human granulosa cells. Fertil. Steril..

[12] Rodgers R.J., Irving-Rodgers H.F. (2010). Morphological classification of bovine ovarian follicles. Reproduction.

[13] Hughes F.M., Gorospe W.C. (1991). Biochemical identification of apoptosis (programmed cell death) in granulosa cells: Evidence for a potential mechanism underlying follicular atresia. Endocrinology.

[14] Hussein M.R. (2005). Apoptosis in the ovary: Molecular mechanisms. Hum. Reprod. Update.

[15] Clark L.J., Irving-Rodgers H.F., Dharmarajan A.M., Rodgers R.J. (2004). Theca interna: The other side of bovine follicular atresia. Biol. Reprod..

[16] Maillet G., Benhaim A., Mittre H., Feral C. (2003). Involvement of theca cells and steroids in the regulation of granulosa cell apoptosis in rabbit preovulatory follicles. Reproduction.

[17] Palumbo A., Yeh J. (1994). In situ localization of apoptosis in the rat ovary during follicular atresia. Biol. Reprod..

[18] Morita Y., Tilly J.L. (1999). Oocyte apoptosis: Like sand through an hourglass. Dev. Biol..

[19] Fan X., Chuva de Sousa Lopes S.M. (2021). Molecular makeup of the human adult ovary. Opin. Endocrine. Metab. Res..

[20] Young J.M., McNeilly A.S. (2010). Theca: The forgotten cell of the ovarian follicle. Reproduction.

[21] Fan X., Bialecka M., Moustakas I., Lam E., Torrens-Juaneda V., Borggreven N.V., Trouw L., Louwe L.A., Pilgram G.S.K., Mei H. (2019). Single-cell reconstruction of follicular remodeling in the human adult ovary. Nat. Commun..

[22] Man L., Lustgarten-Guahmich N., Kallinos E., Redhead-Laconte Z., Liu S., Schattman B., Redmond D., Hancock K., Zaninovic N., Schattman G. (2020). Comparison of Human Antral Follicles of Xenograft versus Ovarian Origin Reveals Disparate Molecular Signatures. Cell. Rep..

[23] Liu T., Huang Y., Lin H. (2021). Estrogen disorders: Interpreting the abnormal regulation of aromatase in granulosa cells (Review). Int. J. Mol. Med..

[24] Themmen A.P. (2005). Anti-Mullerian hormone: Its role in follicular growth initiation and survival and as an ovarian reserve marker. J. Natl. Cancer Inst. Monogr..

[25] Walters K.A., Allan C.M., Handelsman D.J. (2008). Androgen actions and the ovary. Biol. Reprod..

[26] Richards J.S., Ren Y.A., Candelaria N., Adams J.E., Rajkovic A. (2018). Ovarian Follicular Theca Cell Recruitment, Differentiation, and Impact on Fertility: 2017 Update. Endocr. Rev..

[27] Rabahi F., Brule S., Sirois J., Beckers J.F., Silversides D.W., Lussier J.G. (1999). High expression of bovine alpha glutathione S-transferase (GSTA1, GSTA2) subunits is mainly associated with steroidogenically active cells and regulated by gonadotropins in bovine ovarian follicles. Endocrinology.

[28] Gordon S., Pluddemann A. (2018). Macrophage Clearance of Apoptotic Cells: A Critical Assessment. Front. Immunol..

[29] Marion G.B., Gier H.T., Choudary J.B. (1968). Micromorphology of the bovine ovarian follicular system. J. Anim. Sci..

[30] Garcia R., Ballesteros L.M., Hernandez-Perez O., Rosales A.M., Espinosa R., Soto H., Diaz de Leon L., Rosado A. (1997). Metalloproteinase activity during growth, maturation and atresia in the ovarian follicles of the goat. Anim. Reprod. Sci..

[31] Byskov A.G. (1974). Cell kinetic studies of follicular atresia in the mouse ovary. J. Reprod. Fertil..

[32] Hayashi K.G., Ushizawa K., Hosoe M., Takahashi T. (2010). Differential genome-wide gene expression profiling of bovine largest and second-largest follicles: Identification of genes associated with growth of dominant follicles. Reprod. Biol. Endocrinol..

[33] Mlodawska W., Slomczynska M. (2010). Immunohistochemical localization of aromatase during the development and atresia of ovarian follicles in prepubertal horses. Theriogenology.

[34] Inoue S., Watanabe H., Saito H., Hiroi M., Tonosaki A. (2000). Elimination of atretic follicles from the mouse ovary: A TEM and immunohistochemical study in mice. J. Anat..

[35] Payne A.H., Hales D.B. (2004). Overview of steroidogenic enzymes in the pathway from cholesterol to active steroid hormones. Endocr. Rev..

[36] Massett M.P., Bywaters B.C., Gibbs H.C., Trzeciakowski J.P., Padgham S., Chen J., Rivera G., Yeh A.T., Milewicz D.M., Trache A. (2020). Loss of smooth muscle alpha-actin effects on mechanosensing and cell-matrix adhesions. Exp. Biol. Med..

[37] Ruiz-Zapata A.M., Heinz A., Kerkhof M.H., van de Westerlo-van Rijt C., Schmelzer C.E.H., Stoop R., Kluivers K.B., Oosterwijk E. (2020). Extracellular Matrix Stiffness and Composition Regulate the Myofibroblast Differentiation of Vaginal Fibroblasts. Int. J. Mol. Sci..

[38] Kinnear H.M., Tomaszewski C.E., Chang F.L., Moravek M.B., Xu M., Padmanabhan V., Shikanov A. (2020). The ovarian stroma as a new frontier. Reproduction.

[39] Kasuya K. (1997). Elimination of apoptotic granulosa cells by intact granulosa cells and macrophages in atretic mature follicles of the guinea pig ovary. Arch. Histol. Cytol..

[40] Lichtnekert J., Kawakami T., Parks W.C., Duffield J.S. (2013). Changes in macrophage phenotype as the immune response evolves. Curr. Opin. Pharmacol..

[41] Tarique A.A., Logan J., Thomas E., Holt P.G., Sly P.D., Fantino E. (2015). Phenotypic, functional, and plasticity features of classical and alternatively activated human macrophages. Am. J. Respir. Cell. Mol. Biol..

