# Supplementary material for: Classification of Atretic Small Antral Follicles in the Human Ovary
Source: Int J Mol Sci. 2023 Nov 28;24(23):16846. doi: 10.3390/ijms242316846 (PMC10706134; doi:10.3390/ijms242316846)
Supplement: Supplementary file 1 [file ijms-24-16846-s001.zip › ijms-2742256-supplementary.pdf]

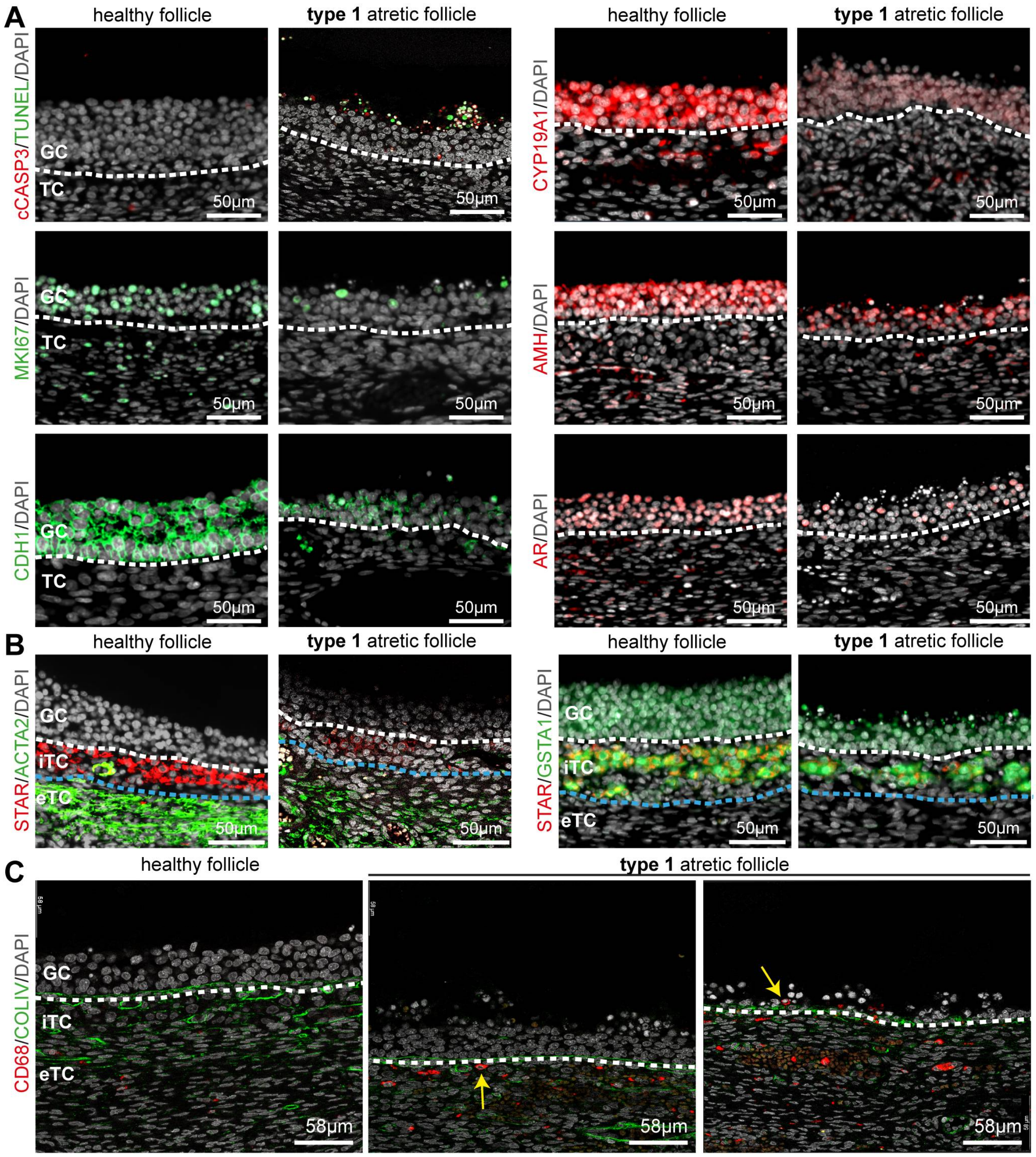

Figure S1. Granulosa and theca cells in healthy small antral follicles and type 1 atretic follicles from transmasculine donors. (A) Immunofluorescence for cCASP3, TUNEL, MIK67, CYP19A1, AMH, AR and CDH1 in healthy small antral follicles and type 1 atretic follicles. White dashed line depicts the basement membrane. White dashed line depicts the basement membrane between granulosa cells (GC) and theca cells (TC). (B) Immuno-fluorescence for STAR and ACTA2 (left panels) and STAR and GSTA1 (right panels) in healthy small antral follicles and type 1 atretic follicles. White dashed line depicts the basement membrane between GC and internal theca cells (iTC); blue dashed line depicts the border between iTCs and external theca cells (eTC). (C) Immunofluorescence for CD68 and COLIV in healthy small antral follicles and type 1 atretic follicles. White dashed line depicts the basement membrane. Yellow arrows point to CD68+ macrophages.

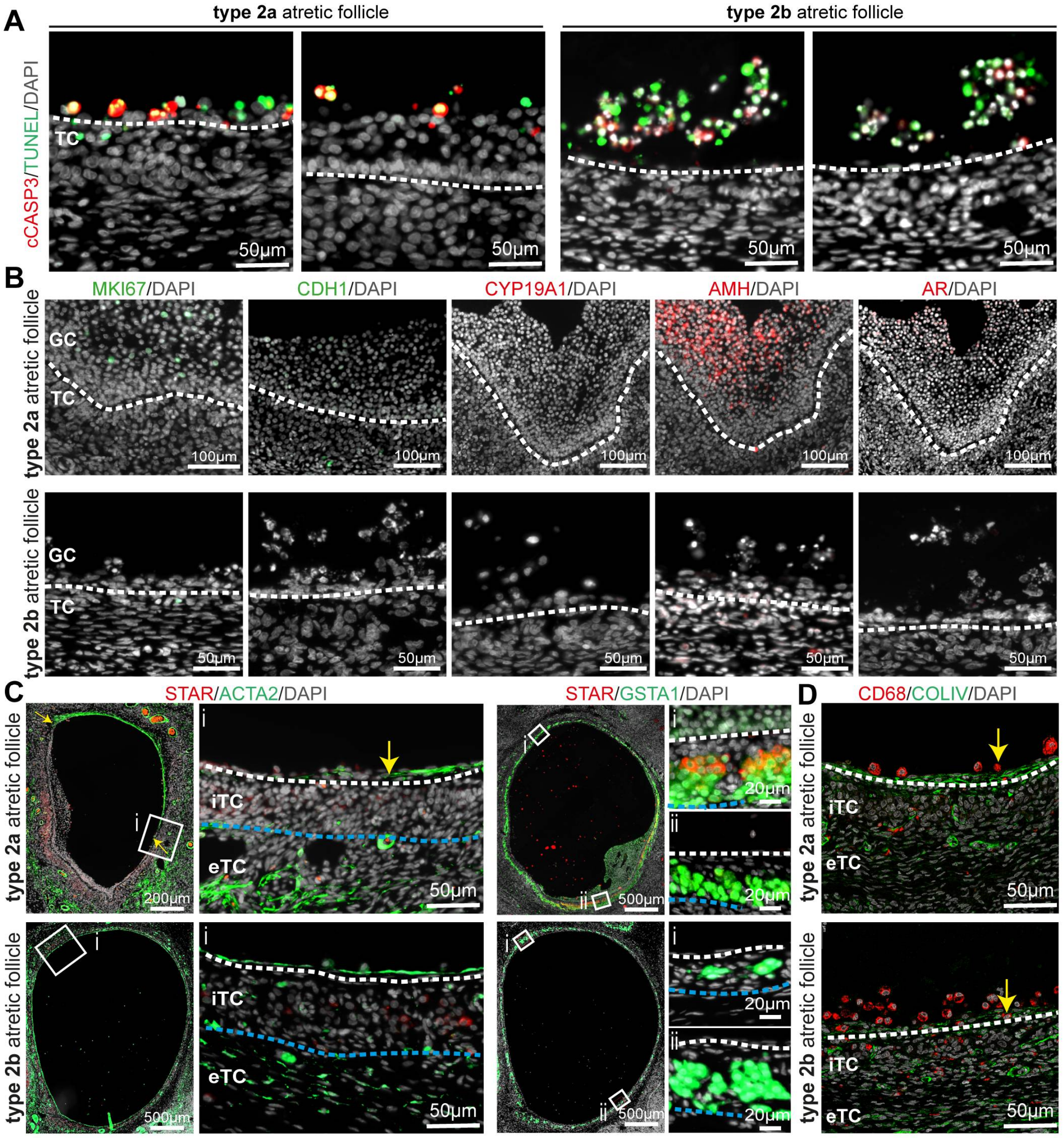

Figure S2. Type 2 atretic follicles in human ovaries from trans masculine donors. (A) Immunofluorescence for cCASP3 and TUNEL in type 2 atretic follicles. White dashed line depicts the basement membrane between granulosa cells (GC) and theca cells (TC). (B) Immunofluorescence for MKI67, CDH1, CYP19A1, AR and AMH in type 2 atretic follicles. White dashed line depicts the basement membrane. (C) Immunofluorescence for STAR and ACTA2 (left panels) and STAR and GSTA1 (right panels) in healthy small antral fol-licles and type 2 atretic follicles. In the magnified areas: yellow arrow points to the transi-tion between ACTA2- to ACTA2+ area bordering the antral cavity; white dashed line de-picts the basement membrane between GC and internal theca cells (iTC); blue dashed line depicts the border between iTCs and external theca cells (eTC). (D) Immunofluorescence for CD68 and COLIV in healthy small antral follicles and type 2 atretic follicles. White dashed line depicts the basement membrane. Yellow arrows point to CD68+ macrophag-es.

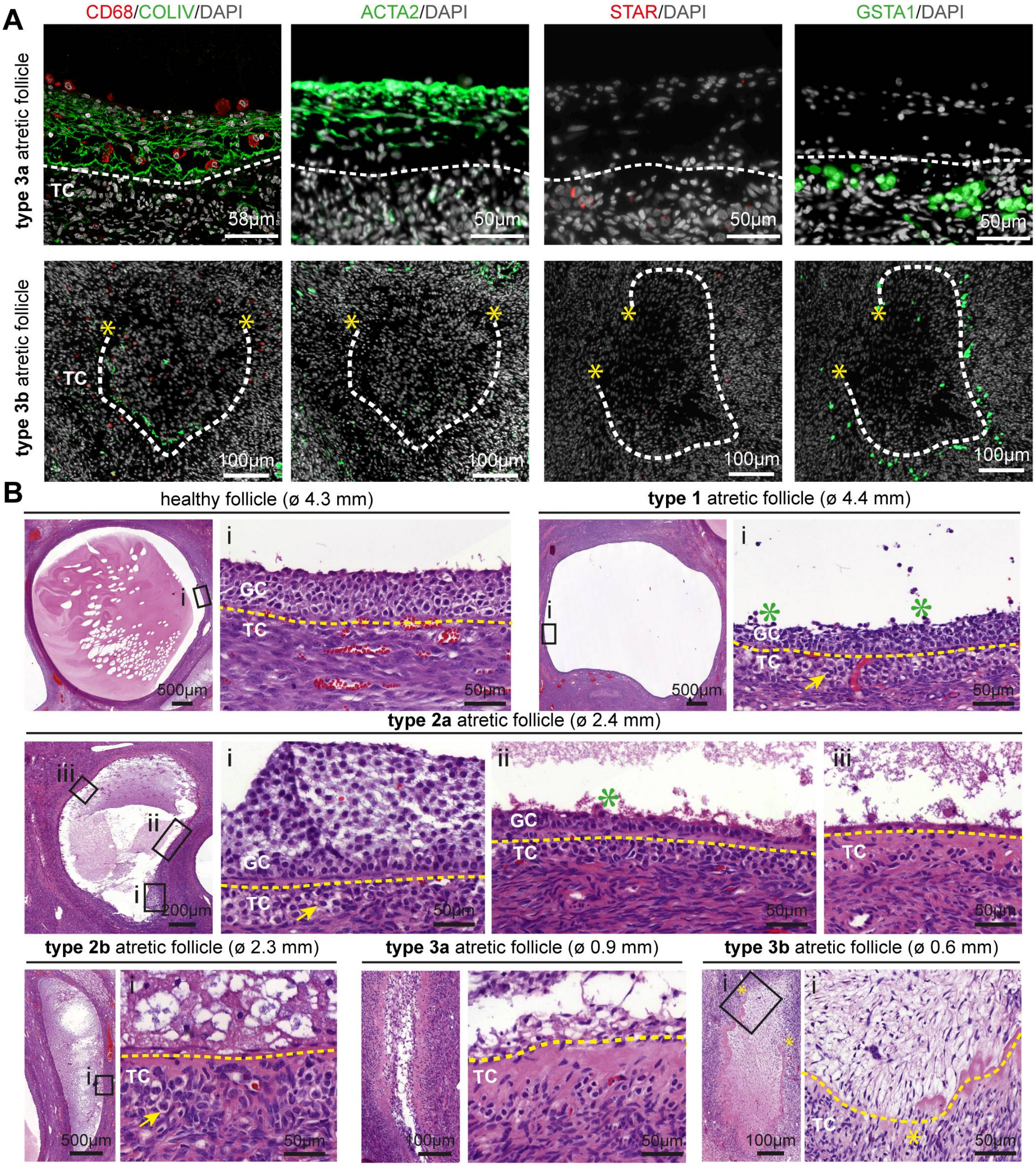

Figure S3. Type 3 atretic follicles in human ovaries from trans masculine donors and characterisation of small antral follicles from a BRCA1 carrier donor. (A) Immunofluo-rescence for CD68, COLIV, ACTA2, STAR and GSTA1 in type 3 atretic follicles from trans masculine donors. White dashed line marks the basement membrane bordering with the-ca cells (TC). Yellow asterisks mark the place where the basement membrane was rup-tured. (B) Histological images of healthy small antral follicle, type 1, type 2 and type 3 atretic follicle from a BRCA1-carrier ovary. Depicted areas contain granulosa cells (GC) and TC. In the magnified hematoxylin-eosin-stained areas: yellow arrow points to a TC with hyaline area; green asterisks in the antral cavity mark GC showing pyknotic nuclei; yellow dashed line marks the basement membrane; and yellow asterisks mark the place where the basement membrane was ruptured.

Table S1. Data of individuals that donated ovary tissue for this study

Donor ID	Age (years)	Type of gender affirming hormone therapy	Duration of gender affirming hormone therapy (months)	Gender identity	(Risk of) Disease
Donor 1	18	Sustanon	34	trans masculine	N/A
Donor 2	18	Sustanon	22	trans masculine	N/A
Donor 3	19	Sustanon	24	trans masculine	N/A
Donor 4	32	Nebido	20	trans masculine	N/A
Donor 5	19	Sustanon	24	trans masculine	N/A
Donor 6	20	Sustanon	30	trans masculine	N/A
Donor 7	36	Sustanon	21	trans masculine	N/A
Donor 8	24	Androgel	35	trans masculine	N/A
Donor 9	23	Sustanon	24	trans masculine	N/A
Donor 10	20	Sustanon	38	trans masculine	N/A
Donor 11	30	Sustanon	22	trans masculine	N/A
Donor 12	22	Sustanon	57	trans masculine	N/A
Donor 13	18	Sustanon	27	trans masculine	N/A
Donor 14	18	Sustanon	22	trans masculine	N/A
Donor 15	28	Sustanon	18	trans masculine	N/A
Donor 16	25	Sustanon	28	trans masculine	N/A
Donor 17	20	Nebido	35	trans masculine	N/A
Donor 18	26	Sustanon	42	trans masculine	N/A
Donor 19	23	Nebido	81	trans masculine	N/A
Donor 20	21	Androgel	41	trans masculine	N/A
Donor 21	20	Nebido	31	trans masculine	N/A
Donor 22	21	Nebido	45	trans masculine	N/A
Donor 23	24	Nebido	42	trans masculine	N/A
Donor 24	26	Sustanon	41	trans masculine	N/A
Donor 25	25	Sustanon	43	trans masculine	N/A
Donor 26	28	Sustanon	26	trans masculine	N/A
Donor 27	26	Androgel	45	trans masculine	N/A
Donor 28	45	N/A	N/A	cis female	*BRCA1* gene mutation
Donor 29	30	N/A	N/A	cis female	brain tumor
Donor 30	19	N/A	N/A	cis female	rhabdomyosarcom a right thigh
Donor 31	33	N/A	N/A	cis female	beta thalassemia major
Donor 32	22	N/A	N/A	cis female	breast carcinoma
Donor 33	30	N/A	N/A	cis female	cervical carcinoma
Donor 34	34	N/A	N/A	cis female	sarcoma lower leg

Table S2. Characteristics of atretic small antral follicles in our study and various reported mammalian species

Our study	Bovine (Marion, 1968)	Bovine (Rodgers, 2001)	Goat (Garcia,1997)	Rat (Osman,1985)	Mouse (Byskov, 1974)	Guinea pigs (Wei, 2010)
**healthy:** 3-10 layers of tightly-packed granulosa cells, several internal theca cells parallel to the intact basement membrane.	**healthy:** 3-10 layers of tightly-packed granulosa cells, rich capillaries, intact basement membrane.	**healthy:** 1-8 layers of uniform granulosa cells, without pyknotic nuclei.	**non-atretic:** translucent appearance, extensive vascularisation and a regular, continuous granulosa cell layer.	**non-atretic:** intact granulosa cell, round oocyte.	**non-atretic:** well-defined granulosa cells, no leucocytes, presence of cavity in follicles.	
**type 1:** pyknotic granulosa cells appear, internal theca cells become rounded (hyaline), basement membrane remains intact, but thickens.		**antral atresia-early:** several pyknotic nuclei in the most antral layers	**stage I:** loss of translucency, vascularization, discontinuous granulosa cell layer.	**stage Ia:** granulosa wall shrinkage, scattered degenerative changes.	**stage I:** up to 20% pyknotic granulosa cells, fragmented nuclei, intact basement membrane, persistent follicular cavity.	**stage I:** connections between granulosa cells became loose, pyknotic granulosa cells and apoptotic bodies observed.
**type 2:** degenerating granulosa cells and basement membrane not intact.	**early atretic:** loose granulosa cells, lost membrana propria, disorganized granulosum basal layer, round internal theca cells.	**antral atresia-late:** numerous pyknotic nuclei in the antrum, the internal theca cell layer well vascularized.			**stage II:** numerous pyknotic nuclei, leucocytes among granulosa cells, disrupted basement membrane, larger cavity than stage I.	**stage II:** few healthy granulosa cells, mass of dead granulosa cells scattered throughout the antrum.
**type 2a:** numerous pyknotic granulosa cells, rounded (hyaline) internal theca cells, basement membrane not intact.	**definite atretic:** advanced regression in collapsing, contracting, cystic, or luteinized-cystic maner.		**stage II:** grayish appearance, poor vascularization, clear interruptions of the granulosa cell layer.			
**type 2b:** all granulosa cells detach from completely cracked basement membrane,			**stage III:** gray dull opaque aspect, few blood vessels, extensive detachments of the granulosa layer.	**stage Ib:** whole granulosa layer degenerates, nuclear fragments in antrum's edge.		

abundant macrophages in the cavity.				**stage IIa:** degenerating oocyte within cumulus cell envelope or remnants.		
**type 3:** reduced follicular size, connective tissue fills the antrum, oocyte degenerates.<br><br>**type 3a:** connective tissue with macrophages, internal thecal cells elongate and are perpendicular to the thick basement membrane.<br><br>**type 3b:** connective tissue with macrophages fills the follicular cavity	**late atretic:** reduced size, disorganized cell layers, a fibrous antrum, and hyalinized theca layers.			**stage IIb:** oocyte 'naked' in the antrum, few nuclear particles.	**stage III:** <5% pyknotic granulosa cell nuclei, leucocytes among granulosa cells, disrupted basement membrane, no cavity observed.	**stage III:** dead granulosa cells were eliminated, antrum was occupied by differentiated cells from the theca layer.<br><br>**stage IV:** the oocyte degenerated, the atrophic follicles were mainly composed of hypertrophic theca cells.
**not observed**		**basal atresia:** the most basal layer of granulosa cells degenerates first, whereas the granulosa cells closer to antrum remained predominantly healthy.				
